# Circulating tumor DNA tumor fraction as a biomarker in refractory metastatic colorectal cancer: findings from the CAVE-2 GOIM trial

**DOI:** 10.1016/j.jlb.2026.100504

**Published:** 2026-09-17

**Authors:** Davide Ciardiello, Giulia Martini, Luca Boscolo Bielo, Gloria Pellizzari, Filippo Pietrantonio, Paola Andena, Silvia Marchesi, Salvatore Pisconti, Claudia Nisi, Giampaolo Tortora, Lisa Salvatore, Andrea Sartore-Bianchi, Salvatore Siena, Federica Papaccio, Marco Messina, Elena Ongaro, Alberto Zaniboni, Carmine Pinto, Lorenzo Antonuzzo, Antonio Avallone, Nicola Normanno, Giuseppe Santabarbara, Maria Giulia Zampino, Alessandra Pia D'Agata, Rossana Berardi, Alessio Cogoni, Claudio Lotesoriere, Tiziana Pia Latiano, Carminia Maria Della Corte, Nicola Fazio, Giuseppe Curigliano, Roberto Bordonaro, Teresa Troiani, Ferdinando De Vita, Erika Martinelli, Fortunato Ciardiello, Stefania Napolitano

**Affiliations:** a Division of Gastrointestinal Medical Oncology and Neuroendocrine Tumors, European Institute of Oncology, IEO, IRCCS, Milano, Italy; b Department of Precision Medicine, University of Campania Luigi Vanvitelli, Naples, Italy; c Division of New Drugs and Early Drug Development for Innovative Therapies, European Institute of Oncology, IRCCS, Milano, Italy; d Department of Oncology and Hemato-Oncology, University of Milano, Milano, Italy; e Department of Medical Oncology, Fondazione IRCCS Istituto Nazionale dei Tumori, Milano, Italy; f Medical Oncology Unit, San Giuseppe Moscati Hospital, Statte, Italy; g Medical Oncology, Università Cattolica del Sacro Cuore, Rome, Italy; h Medical Oncology, Comprehensive Cancer Center, Fondazione Policlinico Universitario Agostino Gemelli, IRCCS, Rome, Italy; i Department of Hematology, Oncology and Molecular Medicine, Grande Ospedale Metropolitano Niguarda, Milano, Italy; j Department of Medicine, Surgery, and Dentistry, Scuola Medica Salernitana, University of Salerno, Baronissi, 84081, Italy; k Medical Oncology Unit, A.R.N.A.S. Ospedali Civico di Cristina Benfratelli (PA), Palermo, 90127, Italy; l Unit of Medical Oncology and Cancer Prevention, Department of Medical Oncology, Centro di Riferimento Oncologico di Aviano (CRO), IRCCS, Aviano, Italy; m Medical Oncology Unit, Fondazione Poliambulanza, Brescia, Italy; n Medical Oncology Unit, Comprehensive Cancer Centre, AUSL-IRCCS di Reggio Emilia, Reggio Emilia, Italy; o Department of Experimental and Clinical Medicine, University of Florence, Italy; p Medical Oncology Unit, Careggi University Hospital, Italy; q Experimental Clinical Abdominal Oncology Unit, Istituto Nazionale Tumori-IRCCS-Fondazione G. Pascale, Naples, Italy; r Center for Advanced Molecular Diagnostics in Oncology, Fondazione Policlinico Universitario Agostino Gemelli IRCCS, Roma, Italy; s Medical Oncology Unit, San Giuseppe Moscati Hospital, Avellino, Italy; t Department of Medical Oncology, Marche Polytechnic University, Ancona, Italy; u Medical Oncology Unit, University Hospital (AOU) of Sassari, Sassari, Italy; v Medical Oncology Unit, National Institute of Gastroenterology, IRCCS de Bellis Research Hospital, Castellana Grotte, Italy; w Medical Oncology Unit, Fondazione IRCCS Casa Sollievo della Sofferenza, San Giovanni Rotondo, Italy; x Medical Oncology Unit, Azienda Ospedaliera ARNAS Garibaldi, Catania, Italy

**Keywords:** Liquid biopsy, Anti-EGFR rechallenge, Cetuximab, Molecular hyperselection, ctDNA tumor fraction

## Abstract

Liquid biopsy guided anti-EGFR rechallenge therapy is gaining ground as a potential option for patient with refractory metastatic colorectal cancer. However, the identification of potential biomarkers to implement patient's stratification is required. The CAVE-2 GOIM trial investigated the role of rechallenge with cetuximab ± avelumab in patients with circulating tumor DNA (ctDNA) (assessed with the FoundationOne Liquid assay) *RAS/BRAF* wild type (WT) mCRC. Correlation with molecular profile, ctDNA tumor fraction (TF), and clinicopathological characteristics was performed. In patients with “negative” compared with “positive hyperselected” tumors the overall response rate was 12% vs 3%, median progression free survival (mPFS) mPFS 5.6 months (4.4-6.9) vs 3.65 months (2.8-4.8) (HR 0.61, 95%CI 0.41-0.91; P = 0.0155), median overall survival (mOS) 14.5 months (12.4-19.0) vs 11.6 months (8.5-15.6) (HR0.61, 95% CI 0.40-0.93; P = 0.023). Therefore, we explored potential biomarkers in patients with “negative hyperselected” tumors. Remarkably, among the investigated factors at multivariable analysis for PFS and OS only ctDNA TF retained significance. For patients with negative hyperselected tumors with ctDNA TF ≥ 10 compared with patients with ctDNA TF <10% mPFS was 4.8 months (3.4-3.9) vs 5.7 (4.4-6.9) (HR 1.74, 95% CI 1.20-2.53; P = 0.00382) and mOS 11.0 months (8.3-13.0) vs 21.3 months (HR 2.75, 95% CI 1.79-4.23; P = 0.00000418). This data suggests that a higher ctDNA shedding might better refine cancer aggressiveness and metastatic load than tumor burden. Taken together these results highlights the strong prognostic value of ctDNA TF and the potential role for treatment personalization.

## 1 Introduction

Liquid biopsy is gaining ground in the management of metastatic colorectal cancer (mCRC) to capture spatial heterogeneity, monitor response, identify potential mechanism/s of acquired resistance and identify minimal residual disease after curative loco-regional therapies [1]. Recently, it has been shown that biomarker-guided anti-epidermal growth factor receptor (EGFR) rechallenge therapy is a potential therapeutic option in patients with circulating tumor DNA (ctDNA) *RAS/BRAF/EGFR-Extracellular domain (ECD)* wild type (WT) metastatic colorectal cancer (mCRC) [[2], [3], [4]]. The rationale of this therapeutic strategy is that resistant cancer cells might progressively decay after discontinuation of EGFR monoclonal antibodies (mAbs), thus potentially restoring sensitivity to EGFR blockade [5].

Growing evidence support that a broader liquid biopsy-based molecular analysis could allow to identify patients without resistance alterations in *genes* other than *RAS/BRAF/EGFR-ECD* that could benefit from anti-EGFR therapies (namely “negative hyperselected” tumors) both in chemo-naïve population and in the refractory setting [[6], [7], [8], [9]]. However, confirmatory studies are required and even in this molecular selected population the identification of potential biomarkers represents an unmet need.

It has been previously shown that ctDNA tumor fraction (TF) might represent a potential prognostic factor in different tumor types, including mCRC [10]. However, validation in a prospective cohort is still lacking.

In this scenario we conducted a pre-planned exploratory analysis of patients enrolled in the CAVE-2 GOIM (Gruppo Oncologico dell’Italia Meridionale) trial to investigate potential biomarkers involved in anti-EGFR rechallenge therapy and explored the potential role of ctDNA TF [11,12].

## 2 Material and methods

The CAVE-2 GOIM study is an academic randomized phase II trial that evaluated the role of cetuximab plus avelumab compared with cetuximab rechallenge therapy as later-line treatment in patients with molecular selected tumors. At baseline, liquid biopsy-based comprehensive genomic profiling (CGP) using the FoundationOne liquid (F1L) CDx assay was performed (https://www.foundationmedicine.com/test/foundationone-liquid-cdx) [11,12] The test interrogates 324 genes: 309 genes are sequenced with complete exonic (coding) coverage, and selected intronic or non-coding regions are targeted in 15 of these genes. A subset of specific regions in 75 genes are captured with increased sensitivity. The F1L CDx assay allows to detect in selected genes base substitutions, insertions/deletions, copy number variations, amplifications, rearrangements, tumor mutational burden (TMB), microsatellite instability, and TF genomic signature.

Patients with microsatellite stable (MSS) tumors without *RAS/BRAF* clonal alterations were included. The primary endpoint is overall survival (OS). Secondary endpoints include progression free survival (PFS), objective response rate (ORR) and safety. Pre-planned translational analysis includes impact of tumor mutation burden and gene expression signatures on clinical outcomes. Here we present mature results with a longer follow-up. Then we explored potential factors involved in anti-EGFR rechallenge therapy in a multivariable model and assessed the role of ctDNA TF. The study protocol and full-inclusion and exclusion criteria have been previously reported [13].

## 3 Statistical analysis

Categorical variables were reported as frequency counts and proportions, whereas continuous variables were reported as medians and interquartile range (IQR). Molecular hyperselection was based on plasma ctDNA analysis of the following genes, which are involved in resistance to anti-EGFR monoclonal antibodies: *KRAS, NRAS, BRAF, EGFR extracellular domain, PIK3CA exon 20, MAP2K1, AKT1, MET, mutations PTEN deletion, and ERBB2 amplification* (tumors without these pathogenic alterations are subsequently reported as negative hyperselected tumors). Based on previous findings a cut-off of 10 was used to differentiate tumors with high ctDNA burden (ctDNA TF ≥ 10) vs low ctDNA burden (ctDNA <10 or not detected) [10,14,15]. The ORR was defined as the percentage of patients who achieved a complete (CR) or partial response (PR) according to RECIST version 1.1. The disease control rate (DCR) was defined as the percentage of cases that achieved a CR, PR or stable disease as best response. PFS was defined as the time from study initiation to progressive disease or death. OS was determined as the time from the beginning of the therapy to death. In the absence of events, PFS and OS were censored at the time of last follow-up.

Median PFS (mPFS) and median OS (mOS) were estimated using the Kaplan-Meier method with confidence interval (CI) calculated with the Brookmeyer and Crowley method and compared between groups using the log-rank statistics. Hazard ratios (HR) were calculated using the Cox-proportional model. Median follow-up was calculated using the reverse Kaplan-Meier method. Inferential statistical tests were carried out using a two-tailed alpha value of 0.05. Key variables potentially correlated with PFS and OS (P < 0.05) were included in a multivariable Cox regression model. Statistical analyses were conducted using Jamovi Software version 2.7.6 and R Software version 4.3.2. Figures were created using Python version 3.11.13.

## 4 Results

**Fig. 1:**
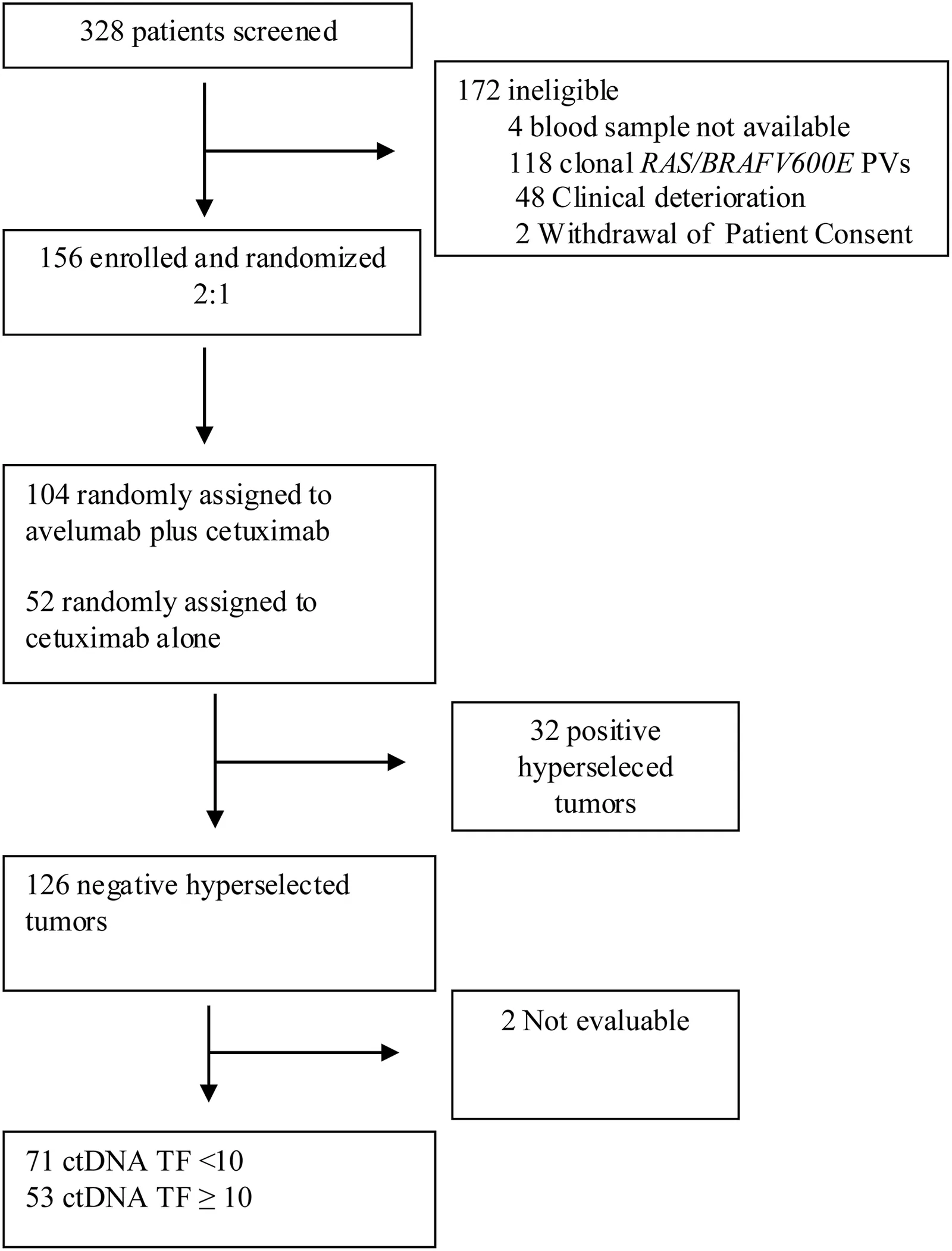
Study diagram. ctDNA TF: circulating tumor DNA tumor fraction; PV: pathogenic variants.

**Fig. 2:**
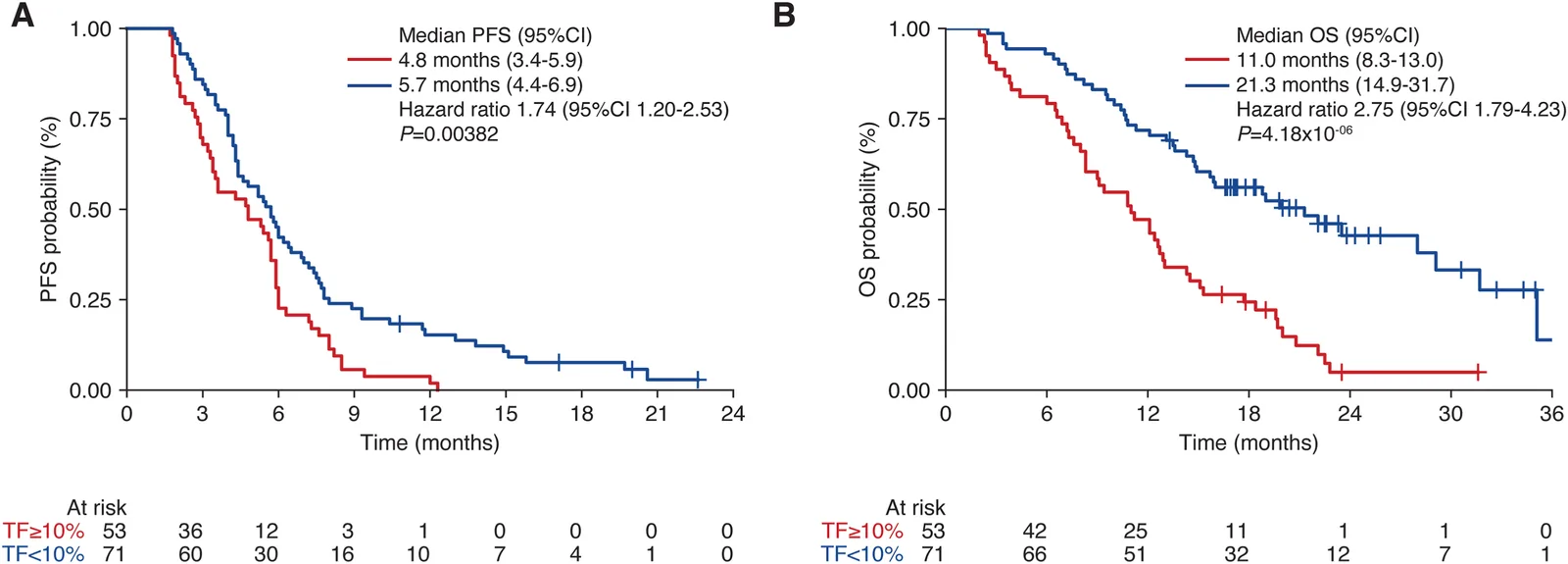
Progression free survival (A) and overall survival (B) according to ctDNA TF. ctDNA TF: circulating tumor DNA tumor fraction; PFS: progression free survival; OS; overall survival.

Overall, 156 patients met the inclusion criteria and were randomly assigned 2:1 to receive cetuximab plus avelumab or cetuximab (Fig. 1). Baseline clinic-pathological characteristics are resumed in Supplementary Table 1. At final data cut-off (30 April 2026) the median follow-up was 27.4 (IQR 22.07-34.27) months. With a longer follow-up, no difference in terms of ORR, disease control rate, mPFS and mOS were observed between the experimental and the control arm (Supplementary Fig. 1). Then, we evaluated the impact of a deeper molecular stratification in response to anti-EGFR rechallenge therapy. In patients with negative compared with positive hyperselected tumors ORR was 12% vs 3%, DCR 68% vs 50%, mPFS 5.6 months (4.4-6.9) vs 3.65 months (2.8-4.8) (HR 0.61, 95%CI 0.41-0.91; P = 0.0155), mOS 14.5 months (12.4-19.0) vs 11.6 months (8.5-15.6) (HR0.61, 95% CI 0.40-0.93; P = 0.023). Since patients with negative hyperselected tumors achieved the highest benefit from cetuximab based rechallenge therapy, we explored potential biomarkers in this subgroup. At univariable analysis the number of previous lines of therapy ≥3 (HR 1.55; 95% CI 1.00-2.41; P = 0.049), the presence of liver metastases (HR 1.66, 95% CI 1.13-2.45; P = 0.01) and ctDNA TF ≥ 10 (HR1.74, 95% CI 1.20-2.53; P = 0.04) was correlated with shorter PFS (Table 1). For OS at univariable analysis the presence of lung metastases was associated with improved survival (HR 0.65, 95% CI 0.42-1.00; P = 0.048), while number of previous lines of therapy ≥3 (HR 1.79, 95% CI 1.11-2.90; P = 0.017) and ctDNA TF ≥ 10 (HR 3.07 95% CI 1.84-5.12; P = 0.001) was associated with shorter survival (Table 1). However, at multivariable analysis for PFS and OS only ctDNA TF retained significance (Table 1). For patients with negative hyperselected tumors with evaluable TF, patients with ctDNA TF ≥ 10 compared with patients with ctDNA TF <10% mPFS was 4.8 months (3.4-5.9) vs 5.7 (4.4-6.9) (HR 1.74, 95% CI 1.20-2.53; P = 0.00382) and mOS 11.0 months (8.3-13.0) vs 21.3 months (14.9–31.7) (HR 2.75, 95% CI 1.79-4.23; P = 0.00000418) (Fig. 2).

**Table 1:** Univariable and multivariable analysis for progression free survival and overall survival in patients with negative hyperselected tumors.

Variable	N	mPFS		mOS	
		Univariable	Multivariable	Univariable	Multivariable
		HR (95%, CI)	HR (95%, CI)	HR (95%, CI)	HR (95%, CI)
**Treatment**					
**cetuximab**	40				
**cetuximab + avelumab**	84	0.84 (0.58-1.23)	0.82 (0.54-1.25)	1.00 (0.64-1.56)	0.82 (0.51-1.33)
		P = 0.376	P = 0.363	P = 0.996	P = 0.423
**Number of previous lines of therapy**					
**2**	97				
**≥3**	27	1.55 (1.00-2.41)	1.61 (0.95-2.71)	1.79 (1.11-2.90)	1.51 (0.87-2.61)
		P = 0.049	P = 0.076	P = 0.017	P = 0.146
**Anti-EGFR free interval**					
**<14.5 months**	62				
**≥14.5 months**	62	0.77 (0.54-1.11)	0.67 (0.44-1.02)	1.04 (0.69-1.58)	0.95 (0.60-1.51)
		P = 0.162	P = 0.060	P = 0.848	P = 0.842
**Gender**					
**Female**	46				
**Male**	78	0.92 (0.64-1.34)	0.84 (0.56-1.26)	1.04 (0.68-1.60)	0.79 (0.49-1.30)
		P = 0.672	P = 0.395	P = 0.851	P = 0.359
**PS**					
**0**	88				
**1**	36	1.14 (0.77-1.69)	1.15 (0.76-1.75)	1.08 (0.68-1.71)	1.08 (0.66-1.77)
		P = 0.504	P = 0.501	P = 0.740	P = 0.760
**Sidedness**					
**Left**	110				
**Right**	14	0.95 (0.48-1.87)	1.02 (0.56-1.85)	0.86 (0.45-1.67)	0.80 (0.40-1.57)
		P = 0.871	P = 0.954	P = 0.665	P = 0.509
**Number of metastatic sites**					
**<3**	80				
**≥3**	44	1.30 (0.89-1.90)	0.99 (0.57-1.72)	1.43 (0.93-2.20)	0.94 (0.46-1.89)
		P = 0.177	P = 0.966	P = 0.099	P = 0.854
**Liver**					
**No**	51				
**Yes**	73	1.66 (1.13-2.45)	1.40 (0.85-2.29)	1.45 (0.93-2.26)	0.92 (0.51-1.66)
		P = 0.01	P = 0.183	P = 0.099	P = 0.783
**Lung**					
**No**	44				
**Yes**	80	0.82 (0.56-1.19)	0.93 (0.58-1.50)	0.65 (0.42-1.00)	0.59 (0.34-1.04)
		P = 0.296	P = 0.762	P = 0.048	P = 0.066
**Peritoneum**					
**No**	100				
**Yes**	24	1.34 (0.85-2.12)	1.55 (0.87-2.75)	1.69 (1.01-2.82)	1.73 (0.88-3.41)
		P = 0.209	P = 0.137	P = 0.046	P = 0.115
**Nodes**					
**No**	73				
**Yes**	51	0.93 (0.64-1.34)	0.85 (0.54-1.33)	1.39 (0.91-2.12)	1.33 (0.75-2.36)
		P = 0.691	P = 0.466	P = 0.125	P = 0.332
**ctDNA TF**					
**<10**	71				
**≥10**	53	1.74 (1.20-2.53)	1.64 (1.04-2.58)	2.75 (1.79-4.23)	3.07 (1.84-5.12)
		P = 0.04	P = 0.033	P = 0.001	P = 0.001

ctDNA TF: circulating tumor DNA tumor fraction; mPFS: median progression free survival; mOS: median overall survival; EGFR: epidermal growth factor; HR: hazard ratio; PS: performance status.

## 5 Discussion

With a longer follow-up findings of the CAVE-2 GOIM study confirm the lack of benefit of adding avelumab to cetuximab rechallenge. Remarkably, the absence of resistance alterations other than *RAS/BRAF* (negative hyperselection) can better stratify patients that could benefit of this treatment strategy.

Different factors have been proposed to implement patient's selection for anti-EGFR rechallenge, including length of the anti-EGFR free interval, sex, the absence of liver metastases [2,16]. Nevertheless, results are still conflicting and none of them has been validated.

To our knowledge, these findings provide for the first time in a prospective cohort of patient enrolled in a clinical trial, evidence on the role of ctDNA TF as a key prognostic factor in refractory mCRC. We among the others, have previously observed that high ctDNA TF was correlated with liver involvement and higher number of metastatic sites [11,17,18]. However, at multivariable analyses ctDNA tumor fraction retained a prognostic role independently of metastatic sites and disease burden. This data suggests that a higher ctDNA shedding is associated with a poor prognosis and might better refine cancer aggressivnes compared with clinical factors and tumor burden.

A retrospective study by Reichert ZR and colleagues investigating the Flatiron dataset, assessed the role of ctDNA TF (assessed with F1L CDx platform) in a cohort of patients with mCRC [10]. The authors observed that a ctDNA TF ≥ 10 was associated with a worse survival. However, the heterogenous study population (inclusion of patients with *RAS/BRAF* WT and mutant tumors; chemo-naïve vs chemo-refractory), the intrinsic nature of a retrospective analysis in a real-world population represents potential limitations. Similar findings were observed in a small cohort of 30 patients with refractory mCRC treated with regorafenib [19]. After adjustment for potential confounding factor, patients with a TF >5% or ≥10% showed a shorter OS.

Taken together these results highlights the strong prognostic value of ctDNA TF and the potential role for treatment personalization. For patients with a lower ctDNA burden and less aggressive disease a less intensive therapy might be considered. In case of a high ctDNA TF is correlated with a worse prognosis, an escalation therapy strategy might be required. By a clinical point of view, for patients with low ctDNA TF rechallenge with single agent anti-EGFR mAbs might spare toxicity while preserving clinical activity. Conversely, patients with elevated ctDNA TF combination of anti-EGFR mAbs with chemotherapy or other therapeutic strategies (trifluridine/tipiracil plus bevacizumab) might be necessary. However, this hypothesis should be interpreted with caution and requires confirmatory studies.

Our investigation has different limitations. First the choice of the optimal cut-off for ctDNA TF was based on previous reports, however, further validation is required [10,14,15]. Second, the absence of a chemotherapy-based regimen does not allow to assess if the addition of chemotherapy to anti-EGFR rechallenge might revert a poor prognostic factor [20,21].

In this scenario, the ROMANCE GOIM trial (NCT07381764) is currently investigating anti-EGFR rechallenge with cetuximab plus irinotecan compared with trifluridine/tipiracil in combination with bevacizumab as third-line therapy, in patients with negative hyperselected tumors. As an exploratory endpoints evaluation of the role of ctDNA TF will be performed.

## Ethics declaration

Written informed consent to take part in the study and to publish the article has been obtained from all participants or their legal representatives. The privacy rights of participants have been observed.

This study included organ or tissue donors. This study includes human biological material and consent was obtained by donors, or their next of kin or legal representatives, for use in this study and for publication of the article. The samples used in this research were not sourced from executed prisoners or prisoners of conscience.

This study was performed in compliance with relevant laws, regulatory frameworks and guidelines where the research took place. This study was approved by the Comitato Etico della Università degli Studi della Campania Luigi Vanvitelli/AOU Luigi Vanvitelli/AORN Ospedale dei Colli di Napoli. (Approval No. 38-22 NP)

The results of this clinical trial and any associated work have been posted in a registry. This clinical trial was registered with number NCT05291156 (Clinical Trial.gov).

## Ethical statement

The study has been conducted in accordance with the principles of the Declaration of Helsinki and has been approved by the Ethics Committee of Università degli Studi della Campania Luigi Vanvitelli—Azienda Ospedaliera Universitaria Luigi Vanvitelli—AORN Ospedale dei Colli and by the ethical committee of all participating centers. The patients/participants provided their written informed consent to participate in this study.

## Data availability statement

The datasets used for this article are available on request from the corresponding author on reasonable request.

## Founding

The CAVE-2 GOIM study was supported by the health care business of Merck KGaA, Darmstadt, Germany for the supply of avelumab and cetuximab and through a research grant that partially covered the costs of the study (CrossRef Funder ID: 10.13039/100009945).

## Conflict of interest

**Davide Ciardiello:** reported receiving travel support from Merck KGaA, Sanofi, and BMS; honoraria for advisory board Bayer, Roche, Merck KgA and Amgen outside the submitted work.

**Giulia Martini:** reported receiving honoraria from Servier, Incyte, and Pierre Fabre outside the submitted work.

**Filippo Pietrantonio:** reported receiving Research funding (to Institution) from Lilly, BMS, Incyte, AstraZeneca, Amgen, Agenus, Rottapharm, Johnson&Johnson, GSK, Tempus, BeOne.

Personal honoraria as an invited speaker from BeOne, Daiichi-Sankyo, Seagen, Astellas, Ipsen, AstraZeneca, Servier, Bayer, Takeda, Johnson&Johnson, BMS, MSD, Amgen, Merck-Serono, Pierre-Fabre, Incyte, AstraZeneca.

Advisory/Consultancy from BMS, MSD, Amgen, Pierre-Fabre, Johnson&Johnson, Servier, Bayer, Takeda, Astellas, GSK, Daiichi-Sankyo, Pfizer, BeOne, Jazz Pharmaceuticals, Incyte, Rottapharm, Merck-Serono, Italfarmaco, Gilead, AstraZeneca, Agenus, Revolution Medicine, 3T Biosciences.

Travel expenses from Amgen, Merck-Serono, Pierre-Fabre, Servier, Astellas, Incyte, Johnson&Johnson.

**Erika Martinelli:** receipt of honoraria or consultation fees for speaker, consultancy or advisory roles: Amgen, Bayer, Eisai, Merck Serono, Pierre Fabre, Roche, Servier, Incyte, Jazz Phc,

Travel grant: AstraZeneca, Pierre Fabre, Bayer, Servier.

**Teresa Troiani:** received travel grants from AstraZeneca and Pierre Fabre and is an advisory board member for AstraZeneca, Bayer, Amgen, Merck, Roche, Sanofi, Servier, and Pierre Fabre.

**Lorenzo Antonuzzo:** received payment or honoraria for lectures and presentation for ASTRA ZENECA, ROCHE, ASTELLAS, NOVARTIS, MSD,BMS,IPSEN, MERK SERONO, AMGEN, BAYER; Support for attending meeting and/or travel grant ASTRA ZENECA, NOVARTIS, IPSEN,MERK SERONO; research founding to my institution: NOVARTIS, ASTRA ZENECA.

**Andrea Sartore-Bianchi:** reported Participation to advisory boards: BAYER, GENMAB, JAZZ PHARMACEUTICAL, MERCK SERONO, PIERRE-FABRE, ROCHE, SERVIER, TAKEDA; Support for attending meeting and/or travel grant: AMGEN, BAYER, PIERRE-FABRE, SERVIER.

**Giampaolo Tortora:** reports funds from the Ministero della Salute (Ricerca Corrente 2022), the AIRC (Investigator Grant number IG26330), Ministero dell’Università e della Ricerca (PRIN 2022 PNRR Prot P2022LN3KS and PRIN 2022 Prot 2022P79F9N), and Agenzia Italiana del Farmaco, Ministero della Salute (J38D19000690001 FIMP,and RF CO-2019-12369662), outside the submitted work; and consulting or advisory role for BMS, AstraZeneca, MSD, Merck, and Servier.

**Lisa Salvatore:** is currently supported by the Associazione Italiana per la Ricerca sul Cancro (AIRC) under My First Grant (MFAG) No. MFAG27367. LS reports consulting or advisory role for Pierre-Fabre, AstraZeneca, Bayer, SERVIER, Merck, Amgen, GSK, Incyte, MSD, Takeda, Nordic Pharma.

**Antonio Avallone:** reported receiving personal fees from Amgen, AstraZeneca, Merck, Sharp & Dohme, Eisai, and Bristol Myers Squibb outside the submitted work.

**Rossana Berardi:** reported consultant/advisory board member for Astellas, Bayer, BMS, Boeringher Ingelheim, EISAI, GSK, Incyte, J&J, Lilly, Lionhealth, Lundbeck.

**Nicola Normanno:** reported receiving honoraria: Thermo Fisher Scientific, Lilly, MSD, Illumina, Merck Serono, Incyte, Biocartis, AstraZeneca, and MSD; consulting or advisory role: Biocartis, AstraZeneca, Bayer, Incyte, Novartis, and Roche; research funding: AstraZeneca (Inst), Biocartis (Inst), Illumina (Inst), Incyte (Inst), Merck Serono (Inst), Qiagen (Inst), Roche (Inst), and Thermo Fisher Scientific (Inst); travel, accommodations, expenses: Merck Serono.

**Giuseppe Santabarbara:** reported participating advisory aoard for Amgen, AstraZeneca, Bayer; travel support from Amgen.

**Carminia Della Corte**: Consultancy/advisory boards: MSD, ASTRA ZENECA, BMS, MERCK, NOVARTIS, PFIZER, AMGEN, PHARMAMAR, DAICHII-SANKYO; Support for attending meeting and/or travel grant: AMGEN, NOVARTIS, PHARMAMAR, MSD, ROCHE, ASTRAZENECA.

**Federica Papaccio:** private research funding from Merck KgA, travel support from Diatech Pharmacogenetics and ESMO Translational Research Fellowship sponsored by Amgen from 2018 to 2020.

**Roberto Bordonaro:** reported receiving honoraria: Novartis, AstraZeneca, Sanofi, Amgen, Roche, Pfizer, Janssen-Cilag, and Bristol Myers Squibb; consulting or advisory role: Novartis, Bayer, AstraZeneca, Sanofi, Amgen, Roche, Pfizer, Janssen-Cilag, and Bristol Myers Squibb; speakers’ bureau: AstraZeneca, Sanofi, Novartis, Bayer, Amgen, Roche, Pfizer, Janssen-Cilag, and Bristol Myers Squibb.

**Nicola Fazio:** reported receiving research funds from and serving on an advisory board for Merck outside the submitted work.

**Giuseppe Curigliano:** reported Grants or contracts from Merck; consulting fees BMS, Roche, Pfizer, Novartis, Lilly, Astra Zeneca, Daichii Sankyo, Merck, Seagen, Ellipsis, Gilead, Menarini; Payment or honoraria forlectures, presentations, speakers bureaus, manuscript writing or educational events from Lilly, Pfizer, Relay, Gilead, Novartis; Support for attending meetings and/or travel from Daichii Sankyo.

**Ferdinando De Vita**: reported Consultant or Advisory Role for Roche, Bayer, BMS, Servier, Lilly, Astellas, MSD, Merck, Astra Zeneca, Dajichi.

**Fortunato Ciardiello:** received institutional research grants from Amgen, Merck KGaA, Merck Sharp & Dohme, Pfizer, Pierre Fabre, Roche, and Servier; and service on advisory boards for Bayer, Merck KGaA, Merck Sharp & Dohme, Pierre Fabre, Roche, and Servier outside the submitted work.

**Stefania Napolitano:** reported receiving personal fees from Novartis and a travel grant from Amgen outside the submitted work.

All other authors have declared no conflicts of interest.

## Abbreviations:

CGP: comprehensive genomic profiling
CI: confidence interval
CR: complete response
ctDNA: circulating tumor DNA
DCR: disease control rate
ECD: extracellular domain
EGFR: epidermal growth factor receptor
F1L: FoundationOne Liquid
GOIM: Gruppo Oncologico dell’Italia Meridionale
HR: hazard ratio
IQR: interquartile range
mAbs: monoclonal antibodies
mCRC: metastatic colorectal cancer
mOS: median overall survival
mPFS: median progression free survival
PR: partial response
ORR: overall response rate
OS: overall survival
PFS: progression free survival
TF: tumor fraction
TMB: tumor mutational burden
WT: wild type
