# Supplementary material for: Circulating tumor DNA tumor fraction as a biomarker in refractory metastatic colorectal cancer: findings from the CAVE-2 GOIM trial
Source: J Liq Biopsy. 2026 Sep 17;14:100504. doi: 10.1016/j.jlb.2026.100504 (PMC13602321; doi:10.1016/j.jlb.2026.100504)
Supplement: Supplementary file 1 [file mmc1.pdf]

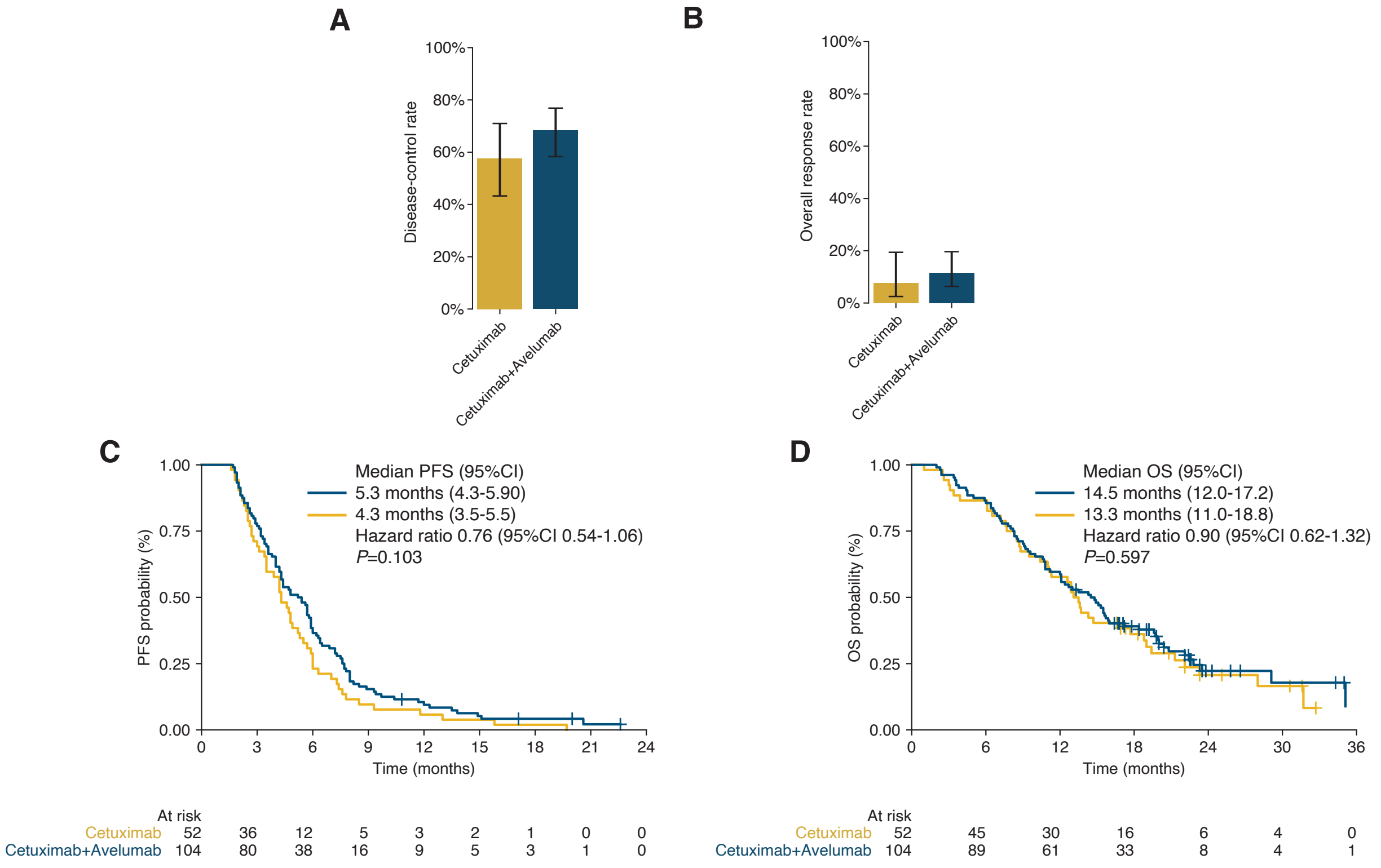
A
Disease-control rate
100%
80%
60%
40%
20%
0%
Cetuximab
Cetuximab+Avelumab
B
Overall response rate
100%
80%
60%
40%
20%
0%
Cetuximab
Cetuximab+Avelumab
C
Median PFS (95%CI)
5.3 months (4.3-5.90)
4.3 months (3.5-5.5)
Hazard ratio 0.76 (95%CI 0.54-1.06)
P=0.103
PFS probability (%)
1.00
0.75
0.50
0.25
0.00
0 3 6 9 12 15 18 21 24
Time (months)
At risk
Cetuximab 52 36 12 5 3 2 1 0 0
Cetuximab+Avelumab 104 80 38 16 9 5 3 1 0
D
Median OS (95%CI)
14.5 months (12.0-17.2)
13.3 months (11.0-18.8)
Hazard ratio 0.90 (95%CI 0.62-1.32)
P=0.597
OS probability (%)
1.00
0.75
0.50
0.25
0.00
0 6 12 18 24 30 36
Time (months)
At risk
Cetuximab 52 45 30 16 6 4 0
Cetuximab+Avelumab 104 89 61 33 8 4 1
