# Supplementary material for: Circulating tumor DNA tumor fraction as a biomarker in refractory metastatic colorectal cancer: findings from the CAVE-2 GOIM trial
Source: J Liq Biopsy. 2026 Sep 17;14:100504. doi: 10.1016/j.jlb.2026.100504 (PMC13602321; doi:10.1016/j.jlb.2026.100504)
Supplement: Supplementary file 2 [file mmc2.pdf]

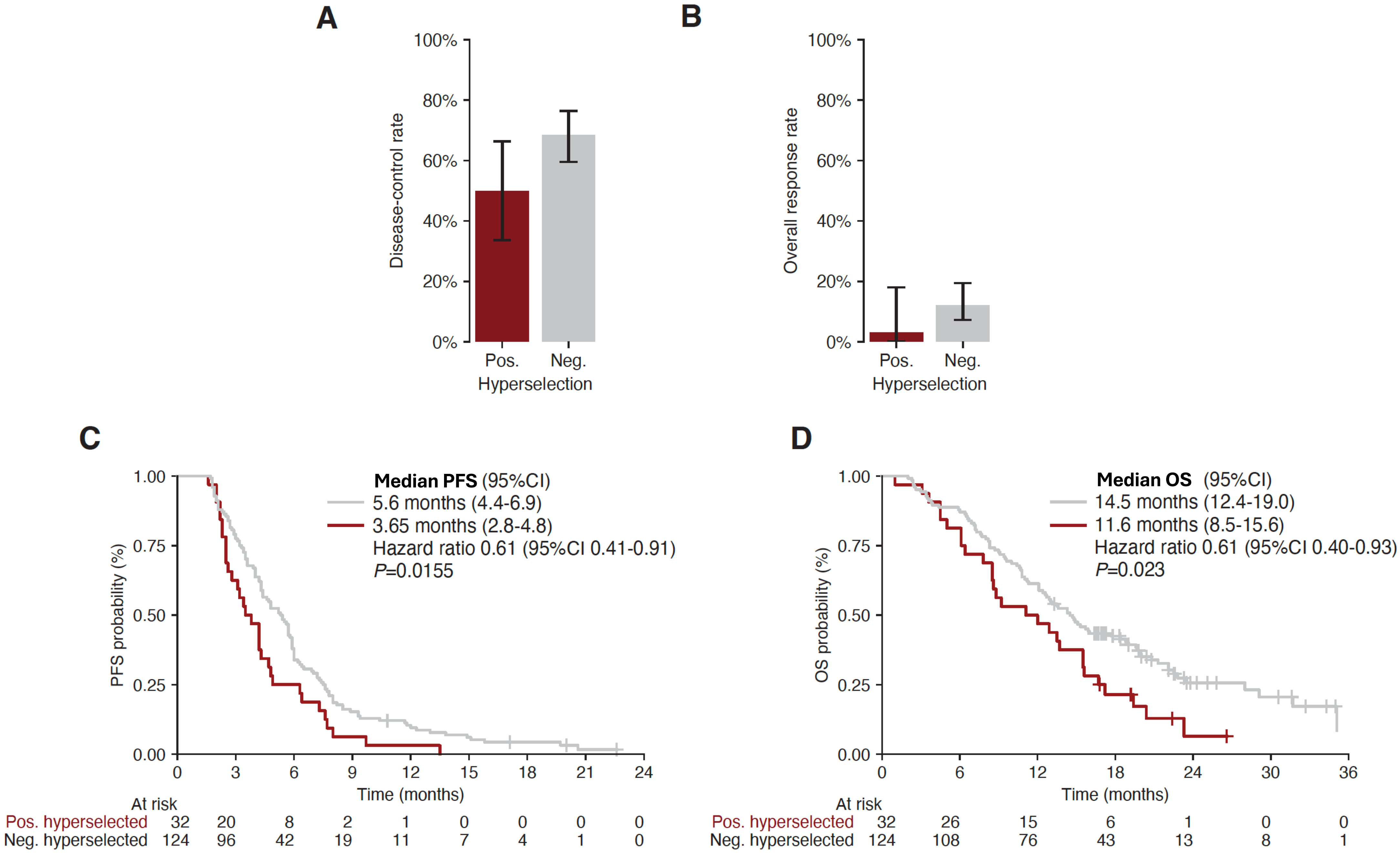
A
100%
80%
60%
40%
20%
0%
Disease-control rate
Pos.
Neg.
Hyperselection
B
100%
80%
60%
40%
20%
0%
Overall response rate
Pos.
Neg.
Hyperselection
C
Median PFS (95%CI)
5.6 months (4.4-6.9)
3.65 months (2.8-4.8)
Hazard ratio 0.61 (95%CI 0.41-0.91)
P=0.0155
1.00
0.75
0.50
0.25
0.00
PFS probability (%)
0 3 6 9 12 15 18 21 24
Time (months)
At risk
Pos. hyperselected 32 20 8 2 1 0 0 0 0
Neg. hyperselected 124 96 42 19 11 7 4 1 0
D
Median OS (95%CI)
14.5 months (12.4-19.0)
11.6 months (8.5-15.6)
Hazard ratio 0.61 (95%CI 0.40-0.93)
P=0.023
1.00
0.75
0.50
0.25
0.00
OS probability (%)
0 6 12 18 24 30 36
Time (months)
At risk
Pos. hyperselected 32 26 15 6 1 0 0
Neg. hyperselected 124 108 76 43 13 8 1
