# Supplementary material for: Circulating tumor DNA tumor fraction as a biomarker in refractory metastatic colorectal cancer: findings from the CAVE-2 GOIM trial
Source: J Liq Biopsy. 2026 Sep 17;14:100504. doi: 10.1016/j.jlb.2026.100504 (PMC13602321; doi:10.1016/j.jlb.2026.100504)
Supplement: Supplementary file 3 [file mmc3.docx]

| Characteristic | Cetuximab+Avelumab | Cetuximab |
| --- | --- | --- |
|  | N = 104 | N = 52 |
| Age at enrollment (IQR) | 64 (57, 72) | 65 (59, 72) |
| Sex |  |  |
| Female | 37 (36%) | 20 (38%) |
| Male | 67 (64%) | 32 (62%) |
| ECOG PS |  |  |
| 0 | 73 (70%) | 38 (73%) |
| 1 | 31 (30%) | 13 (27%) |
| Sidedness |  |  |
| Left | 96 (92%) | 43 (83%) |
| Right | 8 (7.7%) | 9 (17%) |
| Number of metastatic sites |  |  |
| <3 | 71 (68%) | 32 (62%) |
| ≥3 | 33 (32%) | 20 (38%) |
| Liver mets | 60 (58%) | 36 (69%) |
| Lung mets | 68 (65%) | 30 (58%) |
| Peritoneal mets | 24 (23%) | 6 (12%) |
| Nodes mets | 40 (38%) | 21 (40%) |
| Number of previous lines of therapy |  |  |
| 2 | 76 (73%) | 38 (73%) |
| ≥3 | 28 (27%) | 14 (27%) |
| Anti-EGFR free Interval (mean, range) | 14 months  (5-64 months) | 16.5 months (5-46 months) |
| Molecular Hyperselection |  |  |
| Positive Hyperselected | 20 (19%) | 12 (23%) |
| Negative Hyperselected | 84 (81%) | 40 (77%) |
| TMB |  |  |
| High | 22 (21%) | 7 (13%) |
| Low | 81 (79%) | 45 (87%) |
| ctDNA TF |  |  |
| <10 | 50(49%) | 29 (56%) |
| ≥10 | 52 (51%) | 23 (44%) |
| NE | 2 (2%) | 0 (0%) |
